# Characterizing the relationship between health utility and renal function after kidney transplantation in UK and US: a cross-sectional study

**DOI:** 10.1186/1477-7525-10-139

**Published:** 2012-11-23

**Authors:** Luca Neri, Phil McEwan, Karin Sennfält, Kesh Baboolal

**Affiliations:** 1 Dipartimento di Scienze Mediche e di Comunità, Università degli Studi di Milano, Milano, Italy; 2 Center for Outcomes Research, Department of Health Management and Policy, Saint Louis University, Saint Louis, MO, USA; 3 Cardiff Research Consortium, Cardiff, United Kingdom; 4 HEOR Europe, Bristol-Myers Squibb, Rueil-Malmaison, Paris, France; 5 University Hospital of Wales Heath Park, Cardiff, United Kingdom; 6 Dipartimento di Medicina del Lavoro, “L. Devoto”, quarto piano, Via San Barnaba, 8, Milano, Italy

**Keywords:** Kidney transplantation, Chronic kidney disease, Quality-adjusted life years, Kidney function, Self-reported outcomes, I18

## Abstract

**Background:**

Chronic allograft nephropathy (CAN) occurs in a large share of transplant recipients and it is the leading cause of graft loss despite the introduction of new and effective immunosuppressants. The reduction in renal function secondary to immunologic and non-immunologic CAN leads to several complications, including anemia and calcium-phosphorus metabolism imbalance and may be associated to worsening Health-Related Quality of Life. We sought to evaluate the relationship between kidney function and Euro-Qol 5 Dimension Index (EQ-5D_index_) scores after kidney transplantation and evaluate whether cross-cultural differences exist between UK and US.

**Methods:**

This study is a secondary analysis of existing data gathered from two cross-sectional studies. We enrolled 233 and 209 subjects aged 18–74 years who received a kidney transplant in US and UK respectively. For the present analysis we excluded recipients with multiple or multi-organ transplantation, creatinine kinase ≥200 U/L, acute renal failure, and without creatinine assessments in 3 months pre-enrollment leaving 281 subjects overall. The questionnaires were administered independently in the two centers. Both packets included the EQ-5D_index_ and socio-demographic items. We augmented the analytical dataset with information abstracted from clinical charts and administrative records including selected comorbidities and biochemistry test results. We used ordinary least squares and quantile regression adjusted for socio-demographic and clinical characteristics to assess the association between EQ-5D_index_ and severity of chronic kidney disease (CKD).

**Results:**

CKD severity was negatively associated with EQ-5D_index_ in both samples (UK: ρ= −0.20, p=0.02; US: ρ= −0.21, p=0.02). The mean adjusted disutility associated to CKD stage 5 compared to CKD stage 1–2 was Δ= −0.38 in the UK sample, Δ= −0.11 in the US sample and Δ= −0.22 in the whole sample. The adjusted median disutility associated to CKD stage 5 compared to CKD stage 1–2 for the whole sample was 0.18 (p<0.01, quantile regression). Center effect was not statistically significant.

**Conclusions:**

Impaired renal function is associated with reduced health-related quality of life independent of possible confounders, center-effect and analytic framework.

## Introduction

Kidney transplantation (KTX) is the treatment of choice in End Stage Renal Disease. In the EU and the US the number of transplantations performed increased in the last 15 years but waiting lists for transplantation continue to grow because demand exceeds organ supply [1,2]. Graft survival at 1 year is about 98% and 90% in UK and US respectively [3,4]. Unfortunately, graft survival beyond five years has remained unchanged since the 1970s : Chronic allograft nephropathy (CAN) occurs in a large share of transplant recipients and it is the leading cause of late allograft loss despite the introduction of new and effective immunosuppressant [5]. Paradoxically, the widely used calcineurin inhibitors, though effective for immunosuppression, are nephrotoxic, impair glucose homeostasis and thus contribute to late allograft loss and cardiovascular mortality [6-9]. Immunosuppressive regimens that preserve renal function may lead to a reduction in graft failure. Additionally, renal impairment affects several body functions and cause symptoms that may reduce health-related quality of life (HRQOL).

We have recently shown that impaired renal function is associated with reduced health-related quality of life in North-American kidney transplant recipients [10]. However residual confounding due to lack of information concerning clinical history of diabetes and cardiovascular diseases could not be ruled out as an alternative explanation of our previous findings. Additionally, to our knowledge there is no data concerning the possible effect of cross-cultural differences on the association between kidney function and health utilities among European and North-American patients. Since cross-cultural differences in quality of life [11-13] might influence the outcome of cost-effectiveness analyses, it is important to evaluate whether the association of health utilities and renal function is confirmed in the European context. In the present study we evaluated the relationship between health utilities and kidney function after transplantation in patients enrolled in UK and US and whether cross-cultural difference exists between these two countries.

## Methods

### Subjects and procedures

Data was obtained from KTX patients enrolled at the kidney transplant facilities of the Renal Unit at the Cardiff and Wales NHS Trust in Cardiff, UK (n=209) and Saint Louis University Hospital, St. Louis, MO (n=233);

#### Description of US sample and data collection procedures

Patients were identified from the renal departmental database of the Saint Louis University Hospital from January 2008 to June 2008. All adult patients (18–74 years old) with a documented kidney transplantation were asked to answer a self-administered questionnaire during a regular visit at the transplant clinic (n=282). Patients providing informed consent were 233 (82% of the original sample). We excluded patients with multi-organ or multiple transplant, and those with no serum creatinine measurements in the 3 months prior to the interview. Since severe acute health events may affect quality of life and determine transient variation in Glomerular Filtration Rate (GFR) we also excluded patients who underwent major surgery in the months prior to enrollment and those with markers of acute cellular damage (Creatine-Kinase > 200 U/L). The final sample resulted in 137 patients. Information including biochemistry assessments results obtained in the 3 months prior to interview and lifetime medical history was abstracted from clinical charts, transplant coordinators records and electronic medical records. We evaluated the accuracy of data reporting across the 3 different sources by evaluating the agreement of laboratory test results performed on the same date for each subject. Since the agreement was almost perfect (ρ=0.99 for all laboratory test results considered) we merged the information into one common clinical database in order to maximize data completeness. The Saint Louis University Institutional Review Board approved the study protocol.

#### Description of UK sample and data collection procedures

All patients registered in the renal departmental database of the Cardiff and Vale NHS Trust in September 2002 (n=1251) were asked to answer a self-administered questionnaire. Of them 157 were on Continuous Ambulatory Peritonale Dialysis, 268 were on hemodialysis, 115 were on CKD pre-dialysis stage, and 711 received a transplantation. All patients treated at the renal clinic received a postal-survey at their home while those on chronic dialysis at the time of survey were asked to complete the questionnaire during a regular dialysis session . . Patients providing the informed consent were 33.3% of the original sample (29,4% among transplant recipients). For the present analysis we included all adult patients with a documented kidney transplant who completed the survey questionnaire (n=209 of whom 14 were on dialysis after graft failure at the time of survey). and had at least one serum creatinine measurement in the 3 months prior to the interview (n=144). No patient with markers of acute cellular damage in the 3 months prior to survey return date were identified (Creatine-Kinase > 200 U/L). Clinical data including serum creatinine concentrations and medical history were abstracted from the clinical database of the Cardiff and Vale NHS Trust.

### Outcome

Our outcome measure was the Euro-QOL 5-Dimension Index (EQ-5D_index_). Its classification system consists of 5 attributes (Mobility, Self-Care, Usual Activities, Pain/Discomfort, and Anxiety/Depression) defining unique 5-digit health state vectors ranging from 11111 for perfect health to 33333 for the worst possible state of health [14]. Since data from this study may be of particular relevance for cost-effectiveness analyses, we used the tariffs estimated by Shaw et al. to calculate the EQ-5D_index_ score for the US sample [15] and the tariffs developed by Dolan [16] for the UK sample, which allow extensive comparability.

### Predictor and covariates

We obtained clinical information from patients’ charts. We collected all laboratory test results recorded in the 3 month period prior to the date of questionnaire administration (serum creatinine, albumin, hemoglobin, Alanine Transaminase (ALT), Aspartate Transaminase (AST), creatine kinase, glucose, phosphorus and calcium). We estimated Glomerular Filtration Rate (GFR) with the MDRD equation (4-variable) [17]. We classified patients according to renal function with the National Kidney Foundation Chronic Kidney Disease (CKD) staging system [17] (CKD 1–2, eGFR ≥ 60 ml/min/1.73 m2; CKD 3, 60 ml/min/1.73 m2 > eGFR ≥ 30 ml/min/1.73 m2; CKD 4, 30 ml/min/1.73 m2 > eGFR ≥ 15 ml/min/1.73 m2; CKD 5, eGFR < 15 ml/min/1.73m2 or patient on dialysis). Both questionnaire administered in US and UK included a section on socio-demographic characteristics (age, gender, education, ethnicity, employment status). For the US sample, depression was defined by self-reported medical diagnosis in the 12 months prior to the interview or prescription of antidrepssant/anti-anxiety medication as reported in clinical charts. For the UK sample the same comorbidity were defined using ICD9 codes listed in Quan H. et al. [18], and Li, B., et al. [19]. Lifetime diagnoses of diabetes and cardiovascular diseases were abstracted from clinical charts (electronic records and hardcopies) in both samples. Diabetes was defined by ICD-9 (or ICD-10) codes (complicated and uncomplicated as defined in Quan H. et al. [18]) anytime, prescription of insulin or any anti-diabetic drug in the 90 days screening period. Cardio-vascular disease was defined by ICD-9 (or ICD-10) codes anytime in patient’s history (myocardial infarction, congestive heart failure, peripheral vascular diseases and cerebrovascular diseases (Quan H. et al. [18]).

### Analysis

Descriptive statistics were calculated and reported as mean ± standard deviation (or median with interquartile range) for continuous variables and frequency for categorical variables. Differences in socio-demographic and clinical characteristics across CKD stages were tested by χ2 for categorical variables and Analysis of Variance (or Kruskall-Wallis test when appropriate) for continuous variables. We evaluated the unadjusted associations between CKD severity, glucose, AST, hemoglobin, phosphorus, calcium, and Albumin serum concentrations with Spearman’s correlation coefficient. Spearman's correlation was also used to test unadjusted associations between eGFR (or CKD stages) and quality of life.

Ordinary Least Square (OLS) models are traditionally adopted to analyze quality of life data. Under ideal conditions they have attractive properties: the conditional mean is an easy-to-interpret, parsimonious representation of the relationship between a continuous outcome and a predictor variable. Additionally, economic models used in cost-effectiveness analysis adopt adjusted means from OLS models in Quality-Adjusted Life Years (QALY) calculation. For this reason we initially used general linear models to obtain adjusted association estimates. Since EQ-5D_index_ scores were strongly skewed and OLS regression assumptions were not satisfied, we identified the power transformation maximizing model R^2^ with the SAS Proc Transform routine:

$$Q\hat{O}L=\sqrt[{2}]{-\left(QOL\cdot 100\right)+100}.1\text{,}$$

where *Q*$\hat{O}$*L* is the transformed dependent variable of the general linear model and QOL is the raw health utility score.

We specified 2 consecutive steps for the GLM analyses. In the first step we included renal function alone to assess the unadjusted coefficients of association. Variables included in the second step were age, gender, ethnicity, months since transplant, diagnosis of diabetes, hypertension, cardiovascular diseases, anxiety/depression, ALT, AST, glucose levels and center of enrollment. From the second step, we obtained adjusted means for each CKD category (as defined above). Results were back-transformed into the original scale, correcting for back-transformation bias [20]. Significance of trend across CKD categories was assessed by partial Spearman’s correlation including all variables entered at each consecutive step.

Since the distribution of the EQ-5D_index_ score was strongly skewed and a relevant ceiling effect was observed, we used Quantile Regression to evaluate the consistency of the association between renal impairment and HRQOL at the 15th, 30th, 50th, 70th and 85th quantile of the outcome distribution [21] Quantile regression minimizes mean absolute distance at a given quantile, rather than modeling the conditional mean as in standard regression. Quantile regression is robust to departures from ordinary least square assumptions. In quantile regression, a quantile, such as the median, depends on the ranks of the Y values, and not on specific values in the tails of the distribution. Quantile regression was introduced by Koenker and Bassett (1978) [22], for the analysis of linear and non-linear response models. Useful features of quantile regression include (a) the models can be used to characterize the entire conditional distribution of a dependent variable; (b) the resulting estimated coefficients from quantile regression are robust to outlier observations on the dependent variable and violation of normality and homoscedasticity of the error term; (c) the resulting estimators are more efficient than those from OLS in the case that the error term is non-normal; (d) potentially different solutions at different quantiles may be interpreted as differences in the response of the dependent variable to change in the regressors at various points in the conditional distribution of the dependent variable; e) parameter estimates can be interpreted as change in the dependent variable per unit change in the independent variable, allowing direct comparison with OLS parameter estimates. We modeled the relationship of CKD stages (categorical variable) with HRQOL and calculated adjusted medians using parameters estimates obtained with Quantile Regression. We modeled the relationship between eGFR with HRQOL using the raw outcome data. Models have been adjusted for age, gender, education, ethnicity, time since transplant, diagnosis of diabetes, hypertension, cardiovascular diseases, glucose levels and AST. In all models (OLS and Quantile Regression) renal function was included alternatively as a continuous (eGFR) or categorical variable. Center effect was accommodated by including in the regression models an indicator variable denoting the center of enrollment and its interaction term with the main predictor of the analysis. Parameter estimates in all analysis on the continuous predictor refer to a 10 ml/min./1.73 m^2^ change in eGFR.

Blood concentrations of Hemoglobin, Albumin, Calcium and Phosphorus were not considered as confounders in the statistical models since they may be part of the causal pathway linking CKD severity and HRQOL. In order to evaluate whether the association between eGFR and HRQOL was partially independent from the hypothesized mediators we included those variables to the statistical models in a secondary analysis.

We considered *P* values < 0.05 as statistically significant and p<0.10 as marginally significant. We used SAS 9.2® to conduct all the analyses.

### Power considerations

For the main outcome (mean EQ-5D index utility estimate), the pooled sample size would achieve ~80% power of detecting a small effect size of 0.23 in a ANOVA test of 4 groups of similar size. This effect size corresponds to a 0.04 difference across CKD classes, a difference considered clinically significant [16,23-25].

## Results

Demographic and clinical characteristics of study sample are summarized in Table 1. The majority of subjects had mild or moderate CKD (stage 1-2-3, 62.3% and 74.3% in UK and US respectively, p<0.01). The mean eGFR was 39.8 (STD= 22.2) in the UK sample and 53.3 (28.0) in the US sample. UK patients were more likely to have hypertension and had a transplant for a longer time while had higher hemoglobin and calcium levels and were less likely to have diabetes and depression (Table 1). Patients with more advanced CKD had lower hemoglobin (ρ = −0.40; p<0.001), albumin (ρ = −0.35; p<0.001), and calcium (ρ = −0.25; p<0.001) serum concentration while phosphorus levels were directly correlated with the severity of renal disease (ρ = 0.45; p<0.001).

**Table 1:** Socio-demographic and clinical characteristics of study sample

**Characteristics**	**UK Sample** (**N**=**144**)	**US sample** (**N**=**137**)	
	**Mean** (**STD**) **or N (%)**		p*
*CKD stage*			<0.01
CKD stage 1-2	24 (16.8)	51 (37.5)	
CKD stage 3	65 (45.5)	50 (36.8)	
CKD stage 4	37 (25.9)	26 ( 19.1)	
CKD stage 5	17 * (11.9)	9 (6.6)	
*Women*	56 (39.2)	46 (33.3)	0.35
*Age*	52.0 (13.8)	49.1 (12.9)	0.07
*Months since Transplant*	63.7 (40.9)	39.5 (52.8)	<0.01
*Diabetes*	26 (18.2)	46 (33.3)	<0.01
*Cardiovascular Disease (CVD)*	44 (30.1)	31 (22.6)	0.13
*Hypertension (HTN)*	82 (57.3)	40 (29.2)	<0.01
*Depression/Anxiety*	5 (3.5)	15 (11.0)	0.01
*Albumin*	4.1 (0.3)	4.0 (0.4)	0.02
*Hemoglobin*	12.9 (1.6)	12.0 (2.0)	<0.01
*Calcium*	9.8 (0.5)	9.2 (0.9)	<0.01
*Phosphorus*	3.3 (1.1)	3.1 (0.8)	0.08

Characteristics of study samples. * Fourteen subjects were returned to dialysis at the time of survey. * p values based on Χ^2^ or Student’s t-test.

The median EQ-5D_index_ scores in the two samples were 0.73 (UK, IQ range = 0.23) and 0.83 (US, IQ range = 0.20). CKD severity was associated to EQ-5D_index_ score in both samples (UK: ρ= −0.20, p=0.02; US: ρ= 0.21, p=0.02). The mean unadjusted disutility associated to CKD stage 5 compared to CKD stage 1–2 was Δ= −0.22 (p<0.01) in the pooled sample, Δ= −0.25 in the UK sample (p=0.01) and Δ= −0.09 (p=0.04) in the US sample (p values based on 1-way ANOVA, pre-specified linear contrast).

**Figure 1:**
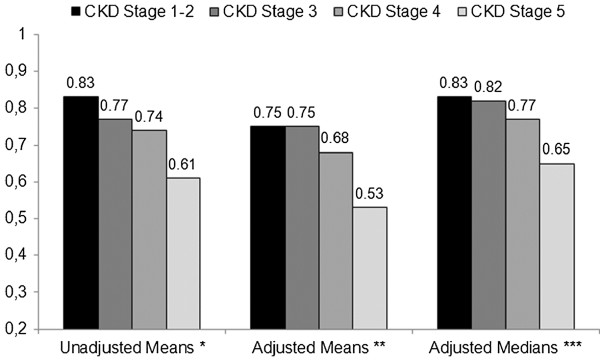
Health Related Quality of Life across CKD stages in the whole sample.

The trend in EQ-5D_index_ across CKD stages evaluated with Spearman’s partial correlation was ρ= −0.18 (p<0.01; pooled sample) , ρ= −0.20 (p<0.01; UK sample) , ρ= −0.18 (p<0.01; US sample) after adjustment for age, gender, center of enrollment, diabetes, cardiovascular diseases, anxiety or depression, hypertension, ALT, AST, months since transplant. The mean adjusted disutility associated to CKD stage 5 compared to CKD stage 1–2 was Δ= −0.38 in the UK sample and Δ= −0.11 in the US sample (Figure 1 and Table 2). After removing the 14 patients on dialysis, only 3 patients remained in CKD stage 5 and the association between CKD severity and HRQOL in the UK sample was not statistically significant (not shown). No significant interaction between age, gender, and eGFR was observed. After including mean serum hemoglobin, albumin and phosphorus in the regression models the association between CKD severity and EQ-5D_index_ was strongly attenuated and lost statistical significance. The interaction term between center of enrollment and CKD severity was not significant in any model.

**Table 2:** Adjusted mean HRQOL scores in study sample

	**CKD stage 1**–**2** (**eGFR** ≥**60**)	**CKD stage 3** (**60** >**eGFR** ≥ **30**)	**CKD stage 4** (**30** >**eGFR** ≥ **15**)	**CKD stage 5** (**eGFR**<**15**)	
**Sample**	**Mean EQ**-**5D**_**index**_				**P**-**value**
**US**	0.83	0.85	0.78	0.72	0.04
**UK**	0.64	0.58	0.49	0.28	0.02

Adjusted mean EQ-5D_index_ scores by kidney function classes. Means estimated with general linear models. Confidence levels refer to partial Spearman’s correlation coefficient. All models have been adjusted for age, gender, months since transplant, diagnosis of diabetes, hypertension, cardiovascular diseases, anxiety/depression and center of enrollment. The interaction term between center and CKD classes was not statistically significant.

**Figure 2:**
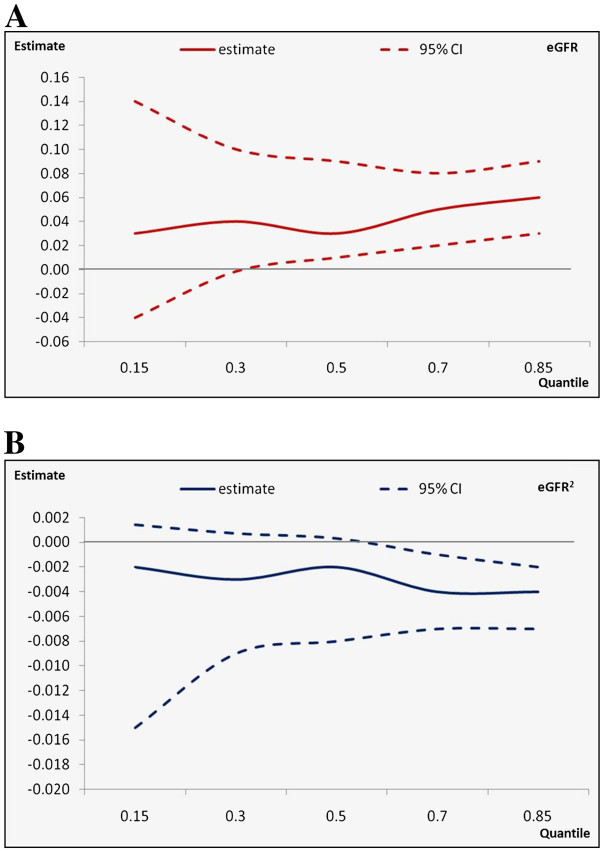
Relationship between estimated Glomerular Filtration Rate and Health Related Quality of Life.

Since the interaction between CKD severity and center of enrollment was not significant, we tested the association between renal impairment and EQ-5D_index_ with quantile regression in the pooled sample. Results from quantile regression are summarized in Figures 1 and 2. The adjusted median disutility associated to CKD stage 5 compared to CKD stage 1–2 for the whole sample was 0.18 (Wald test for trend across categories, p<0.01), slightly smaller than the adjusted mean disutility estimated from GLM (0.22, Wald test for trend across categories, p<0.01) (Figure 1). According to the results obtained evaluating the quantile process (τ being evaluated: 0.15, 0.30, 0.50, 0.70, 0.85) the association between eGFR (entered as a continuous variable) and EQ-5D_index_ was slightly stronger in the upper tail of the outcome distribution. Parameter estimates ranged from 0.02 (τ=0.15) to 0.06 (τ=0.85) and were not significant at τ<0.30 (Figure 2a). In the upper half of the outcome distribution (τ>0.50) the relationship between eGFR and EQ-5D_index_ was non-linear: the coefficient estimate for eGFR^2^ ranged from −0.003 to −0.004 and was statistically significant at the 5% confidence level (Figure 2b). The interaction term between the indicator variable denoting the center of enrollment and eGFR was not significant at any percentile of the outcome variable.

## Discussion

According with previous findings [10,26], we observed a statistically significant association between kidney disease severity and health utilities. This relationship was robust to adjustment for several confounders and was observed in both the US and UK samples. However after including hemoglobin, phosphorus and albumin serum concentrations in the analysis, the association of CKD severity and EQ-5D _index_ was strongly attenuated and was not statistically significant. Since Chronic Kidney Disease is associated with reduced hematopoiesis, accumulation of waste products, mineral metabolism imbalance and chronic inflammation, our results suggest that that the association between HRQOL and CKD could mostly be explained by its related functional impairments. However, hypotheses concerning meditational effects are beyond the scope of our cross-sectional design and should be further tested in longitudinal studies adopting appropriate statistical techniques (i.e. path analysis or structural equation modeling).

We have found no evidence of a moderating center effect on the relationship between HRQOL and CKD severity in both OLS and Quantile regression framework. However the difference across centers in the estimated disutility associated to CKD stage 5 compared to CKD stage 1–2 was large and clinically significant according to proposed thresholds defining minimal clinically important difference [16,23-25] and merits further investigation. Previous research have highlighted possible cross-cultural discrepancies in health-related quality of life [11-13]. However, US preference-based algorithm produces scores with a smaller range than the UK scores, possibly resulting in smaller difference scores and partially contributing to cross-national differences in health utility assessments. Additionally, while all patients in the US sample had a functioning graft, the CKD stage 5 group in UK sample included 14 patients who returned to dialysis after graft loss at the moment of the interview. After removing from the analysis all patients on dialysis after graft failure only 3 patients remained in CKD stage 5 group (UK sample), preventing a statistically stable estimation of association. Whether patients with failed graft should be included in the analysis of health utility after kidney transplant is debatable. Returning to dialysis is the necessary course of events for CKD stage 5 patients if survival is to be significantly prolonged and this choice is part of the natural history of the disease for all patients with severe renal impairment. While it is conceivable that graft failure may impact on patients’ physical and emotional status beyond the effects of glomerular filtration loss, dialysis partially substitutes for bodily functions otherwise insufficient in CKD 5 patients on conservative treatment. Which effect is predominant in transplant patients is a matter of empirical testing. Ideally the HRQOL reported by CKD stage 5 patients on conservative treatment should be contrasted against that of those returned on dialysis. However such a design is particularly challenging since transplant patients on stage 5 spend little time on conservative therapy and are often treated in different locations when they return to dialysis. In the only study indirectly allowing such a comparison there was no apparent differences in general HRQOL and health utility scores between dialysis patients and CKD stage 5 on conservative treatment [26]. However the sample size of these subgroups was very small and replications of such studies would be of great importance.

It is worth noticing that the difference observed across CKD classes is clinically significant in both samples [16,23-25]: the unadjusted and adjusted differences across CKD classes in our sample were equal or larger than the suggested threshold for the minimal clinically important difference in health utility measures. The observed disutility associated with severe CKD corresponded to 40 and 138 days of healthy life lost for every year in CKD stage 5 compared to CKD stage 1–2 in the US and UK sample respectively.

Finally, we used quantile regression to evaluate the consistency of the association between renal impairment and HRQOL at the 15th, 30th, 50th, 70th and 85th quantile of the outcome distribution and to corroborate results from OLS regression. Even though statistically significant, median regression, which is equivalent to a quantile regression estimated for τ=0.50, yielded somewhat smaller disutility estimates compared to general linear models (ordinary least square). The quantile process estimated entering eGFR as a continuous variable indicate that the relationship between eGFR and HRQOL was slightly stronger in the upper tail of the outcome distribution. The difference in CKD-related disutility between the 15^th^ and the 85^th^ percentile of the outcome distribution exceeds the suggested threshold for clinical significance in utility scores, thus indicating that patients with otherwise better conditions suffer a bigger utility penalty form reduced renal function than patient with worse general health conditions. As a consequence our results suggest that unobserved factors might moderate the association of renal function and HRQOL. Several factors has been shown to moderate the relationship between health status and QOL including income [27], social support [28], personality traits [29] and contextual factors [30]. In our sample neither gender nor age moderated the relationship between quality of life and renal function. However our study was not designed to test moderating effects by measured clinical characters and other potentially important factors on the observed relationship.

The strengths of the present study are: i) our data expands the current knowledge on cross-national HRQOL studies; ii) we adjusted for several known correlates of HRQOL in multivariable regression ; iii) we used multiple serum creatinine assessments collected in the 3 month period prior to enrollment to define study groups in order to minimize classification bias; iv) we confirmed the consistency of our results under different analytic frameworks. However, our study presents some limitations. First, we lacked information on possibly important confounders such as type of transplant (i.e. living/deceased donor), immunosuppression regimen, patients’ personality traits. As a consequence residual confounding cannot be ruled out as an alternative explanation of our results. One additional limitation of this study is its correlational design. Additionally data collection was differently performed in the US and UK studies, which may partially bias our cross-national comparison. Further, only a small fraction of UK eligible patients responded to the questionnaire leaving a potential for selection bias possibly limiting the generalizability of our results: our sample had poorer renal function and were more likely to have hypertension and diabetes compared to the general KTX population in UK [31,32]. Finally, cross-sectional studies cannot provide evidence of causality: even though our results are consistent with the hypothesis that the severity of renal disease and CKD-related functional impairments negatively affects patients’ wellbeing, reverse causality cannot be ruled out with our data.

## Conclusions

In the present cross-national cross-sectional study, we observed that a decline in kidney function was associated with worsening health utility estimates following kidney transplantation. This association was robust for adjustment for several established correlates of HRQOL and was confirmed in both UK & US centers. Even though the center effect was not statistically significant, the magnitude of the difference in health disutilities observed in the two centers merits further evaluation. Results from quantile regression confirmed the validity of the association between eGFR and EQ-5D_index_ scores observed under OLS analytic framework and suggests that unobserved factors might moderate this association. Further studies specifically designed to characterize such moderating factors might help identify patients more likely to benefit from kidney function preserving strategies. The present analysis provides further evidence supporting efforts in preserving renal function after kidney transplantation.

## Competing interest

LN and PME received consulting fees from BMS pharmaceuticals. KS is employed at BMS pharmaceuticals. KB has no competing interests.

## Authors' contributions

Study design: LN and PME. Data Analysis: LN. Interpretation of results: LN, PME, KS, KB. First draft: LN. Revision and Final draft: LN, PME, KS, KB. All authors read and approved the final manuscript
